# Interlayer Reinforcement Combined with Fiber Reinforcement for Extruded Lightweight Mortar Elements

**DOI:** 10.3390/ma13214778

**Published:** 2020-10-26

**Authors:** Carla Matthäus, Nadine Kofler, Thomas Kränkel, Daniel Weger, Christoph Gehlen

**Affiliations:** Chair of Materials Science and Testing, Technical University of Munich, Centre for Building Materials, Franz-Langinger-Str. 10, 81245 Munich, Germany; koflernadine@gmail.com (N.K.); thomas.kraenkel@tum.de (T.K.); daniel.weger@tum.de (D.W.); gehlen@tum.de (C.G.)

**Keywords:** additive manufacturing, extrusion, lightweight aggregate mortar, reinforcement, fibers, vertical reinforcement, layer bonding, anisotropy

## Abstract

Lightweight mortar extrusion enables the production of monolithic exterior wall components with improved thermal insulation by installing air chambers and reduced material demand compared to conventional construction techniques. However, without reinforcement, the systems are not capable of bearing high flexural forces and, thus, the application possibilities are limited. Furthermore, the layer bonding is a weak spot in the system. We investigate a reinforcement strategy combining fibers in the mortar matrix with vertically inserted elements to compensate the layer bonding. By implementing fibers in the extruded matrix, the flexural strength can be increased almost threefold parallel to the layers. However, there is still an anisotropy between the layers as fibers are oriented during deposition and the layer bond is still mainly depending on hydration processes. This can be compensated by the vertical insertion of reinforcement elements in the freshly deposited layers. Corrugated wire fibers as well as short steel reinforcement elements were suitable to increase the flexural strength between the layers. As shown, the potential increase in flexural strength could be of a factor six compared to the reference (12 N/mm^2^ instead of 1.9 N/mm^2^). Thus, the presented methods reduce anisotropy in flexural strength due to layered production.

## 1. Introduction

### 1.1. Lightweight Mortar Extrusion

The extrusion of lightweight mortar poses new possibilities in building construction. Structures can be optimized for the building envelope by integrating (a) a material with a low thermal conductivity and (b) the geometrical freedom as well as function integration of the extrusion process. Extruding fine mortar strands, interspaces that might be filled with a loose insulation material or closed hollow air voids can be integrated in the structure without increasing time and effort. This way, the thermal conductivity is further reduced in comparison to a massive element. Furthermore, an efficient structural and thermal design can easily be realized on the basis of a virtually optimized model [1,2,3]. Placing material only where it is needed without formwork leads to a reduction of resource consumption and waste [3]. Furthermore, 3D printing also opens up possibilities for novel infrastructure application.

The lightweight mortar induces less weight into the system than a normal concrete which facilitates the buildability (ability to withstand deformations/failure during the building process) of the material [4]. However, the transmission of loads, especially bending loads, still poses a challenge and requires special material development or reinforcement strategies. The flexural strength of extruded elements is often direction dependent due to the layerwise production. Especially if the contour length is large, the time gap between the deposition of individual layers increases and leads to superficial drying and thus to cold joints (week layer bond) and anisotropy of the material [5,6,7].

Therefore, we aim for a reinforcement technique that compensates the anisotropy and is suitable for integration into the extrusion process.

### 1.2. Reinforcement Systems for Extrusion (State of the Art)

The applicability of 3D-printed elements for structural purposes is currently limited [8,9]. Ideas for an appropriate integration of reinforcement have been listed by [10] and need to be tested with regard to their potential and applicability. These include, among others, external reinforcement systems, insertion of reinforcement bars or tensioning elements in reserved zones created by cavities during 3D-printing [11] using the printed structure as “stay-in-place formwork.” In this paper, we concentrate on reinforcement strategies that limit the geometric freedom as little as possible and are implemented directly in the mortar.

Existing reinforcement systems such as steel rebars, wires, fibers or rovings can conceivably be used for 3D-printed elements. However, they must be compatible with the specific manufacturing process as well as with the shape and geometry of the printed elements and must be able to be integrated into the process [10]. Thus, they limit the geometrical freedom. A technology that poses few challenges during the manufacturing process is the addition of fibers in the concrete matrix. For several decades, fibers have been used in concrete to limit cracks or to reduce the content of reinforcing steel. Fibers made of different materials are available. However, steel fibers are used most frequently [12].

In the literature, the first investigations on fibers for reinforcement of 3D-printed elements can be found, e.g., [12,13,14,15,16,17,18,19]. An orientation of the fibers can be observed during the extrusion process, which can be used deliberately [12,19]. If the flexural strength is tested parallel to the oriented fibers, it can be increased significantly [12]. Likewise, the inlaying of a metal cable during the deposition process lead to good connection between reinforcement and concrete and an increase in flexural strength [20]. However, the flexural strength depends on the test direction [13,21]. This is especially true for elements with a bad layer bonding. Differences in the magnitude from 15% to 50% between prisms sawn out transverse or parallel to the printing direction were found depending on the amount and length of integrated glass fibers [13]. In those cases, the fibers are located in individual layers and are not connecting the layers with each other. The connection between the layers is still merely established due to concrete hydration. Thus, the strength of the layer bonding may either be not affected or even be deteriorated due to increased anisotropy. The layer bond was therefore identified as the weakest zone of the elements [22,23].

The aim is thus to interlock the layers with each other. In order to achieve that, [24] tried to interlock the layers mechanically by specially shaped nozzles. Their investigations on molded concrete led to an increase in flexural strength of up to 26% [24]. Another possibility is to use reinforcement perpendicular to the layers. This can be inserted into the deposited layers in predetermined intervals (see e.g., [25]) or build up simultaneously with the layers (see e.g., [9]). The combination of reinforcement built-up layer by layer with e.g., gas-metal arc welding and molded concrete showed comparable mechanical properties to common steel reinforcement and satisfying bonding between rebars and concrete [9]. However, the integration in the layerwise production of the concrete 3D-printing is a challenge, which has not been satisfactorily solved yet. Weger et al. present the first results on the temperatures that occur during the layerwise welding process and the impact on the properties of cementitious systems [26].

A second option of vertical reinforcement is to connect the layers by insertion of vertical elements. This can be implemented by placing a textile with single vertical fibers between two layers [9] or by inserting individual elements as fibers, nails, screws or rebars. Recently, the nailing of the layers was described in literature [25]. Perrot et al. [25] examine the insertion of straight steel nails through up to 10 freshly deposited mortar strands. Inclined nails led to an increase in flexural strength in the magnitude of 50%. The surface properties of the nails (smooth or rusty) played only a minor role [25]. Furthermore, first studies about the insertion of screws have been conducted [27,28,29]. They found that screwing insertion or the application of a grouting mortar lead to better bond between reinforcement and concrete than direct insertion [27,29]. Thus, the pull-out forces for the reinforcement and the overall mechanical performance increase.

Within the scope of the collaborative research center TRR 277 “Additive Manufacturing in Construction – The Challenge of Large Scale” between Technical University of Munich and Technische Universität Braunschweig (see notes on funding), studies on vertical reinforcement by insertion of fibers and rebars have been conducted. First pre-investigations were conducted by TUM in mid-year 2019 for the project “Extrusion of Near-Nozzle Mixed Concrete –Individually Graded in Density and in Rate of 3D Fiber Reinforcement.” These first results will be presented in this paper.

### 1.3. Objective and Problem Definition

The objective of this paper is to increase the flexural tensile strength of extruded lightweight mortar elements by implementing a combination of two reinforcement systems. Micro steelfibers are implemented in the extruded matrix to increase the flexural strength in layer direction. In order to reduce the direction dependency of the flexural strength due to the weakening layer bond as described in Section 1.2, different types of fibers and reinforcement bars are inserted vertically into the mortar strands (see Section 2.2). The vertically inserted elements are supposed to connect the layers with each other and lead to a uniform transfer of the loads.

## 2. Materials and Methods

### 2.1. Materials for Lightweight Aggregate Mortar

The lightweight mortar mix contains an OPC with a specific surface *A_Blaine,C_* = 5600 cm^2^/g, medium grain size of *d*_50,C_ = 7.9 µm and a specific density of 3.1 kg/dm^3^. The cement was partly replaced by 20 vol.% of limestone powder (LP). For the reference mixture A, we used a rather coarse limestone powder with *A_Blaine,L_* = 3700 cm^2^/g, *d*_50,L_ = 15.7 µm and a specific density of 2.7 kg/dm^3^. Reference B contains a finer limestone powder with *A_Blaine,L2_* = 11,400 cm^2^/g, *d*_50,L2_ = 2.2 µm and a specific density of 2.7 kg/dm^3^. Furthermore, we used silica fume with a specific density of 2.3 kg/dm³ and *d*_50,S_ = 0.15 µm. In order to adjust the flowability of the mixtures, we added a polycarboxylate ether (PCE) based superplasticizer with a solid content of 40% by mass. Besides, a thixotropy enhancing agent and a stabilizer were used to enhance pumpability and buildability [30].

All mixtures had a water/binder ratio of 0.38 by mass and the ratio between cement paste and aggregates was 0.9. The lightweight aggregates are expanded glass granulates with a maximum grain size of 2 mm. For all experiments, their particle size distribution and the water content (mixing and suction water) were kept constant.

### 2.2. Materials of Investigated Reinforcement Systems

**Figure 1:**
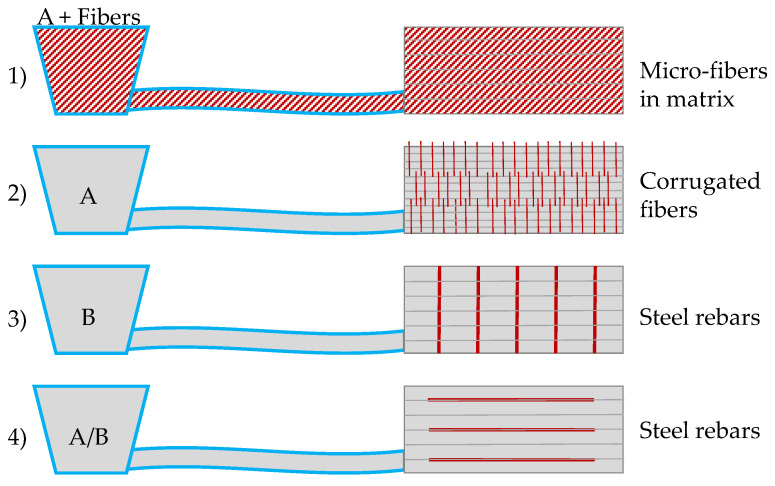
Schematical overview on experiments and reinforcement types.

The lightweight aggregate mortar (described in Section 2.1) was used as a reference material. Based on this, several reinforcement systems were investigated. The aim of this paper is to evaluate the potentials of different reinforcement concepts—namely, micro steelfibers in the matrix as well as vertical and horizontal reinforcement with fibers/rebars—for the extrusion of lightweight mortar and to analyze their mode of action and integration into the printing process. Figure 1 gives an overview on the types of reinforcement and the combination with reference material A or B. The system on the top (1) is based on reference material A but includes fibers in the matrix.

For the fibers that are integrated in the matrix, we used micro steelfibers with a length of 9 mm and a diameter of 0.15 mm (aspect ratio of 60). For the vertical reinforcement, wire fibers as well as reinforcement bars (rebars) were used. We compared corrugated wire fibers with fibers with end hucks. Both had a length of 50 mm and a diameter of 1 mm (aspect ratio of 50). Their tensile strength is 1100 N/mm^2^ and their elastic modulus is 210,000 N/mm^2^. Furthermore, conventional steel rebars (B500A) with a diameter of 6 mm and ribbed glass fiber rebars with a diameter of 5 mm were cut to a length of 200 mm for the vertical insertion into the 3D-printed elements. Last but not least, conventional steel rebars (B500A) with a diameter of 6 mm were also used for horizontal placement between the layers.

### 2.3. Material Preparation

For pre-wetting, the lightweight aggregates were mixed with two thirds of the total water amount for one minute and left to absorb the water for another 4 min before addition of cement, additives and dry admixtures. After a mixing time of 1 min, the remaining water and PCE was added and the mixture was mixed for additional 45 s. The mixing container was scraped in order to remove any powder adhering to the edges. If fibers were added, this was done at that time. Finally, the material was mixed for another 45 s.

### 2.4. Sample Geometries and Fabrication

**Figure 2:**
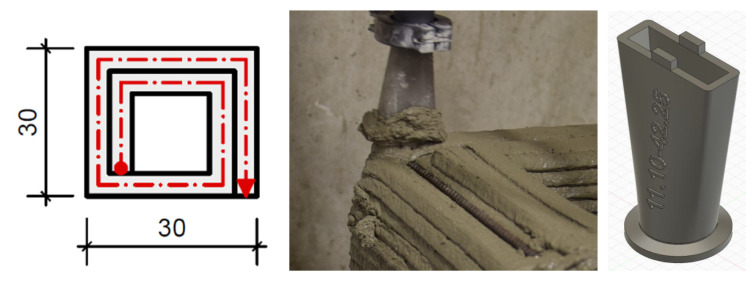
Sample geometriy (**left**), deposition of a layer on top of horizontal reinforcement (**middle**) and nozzle with tongue (**right**).

For each reinforcement concept, simple geometries of lightweight mortar were extruded and reinforced with the respective reinforcement. The 3D-printed test specimens had a square shape with dimensions of 30 cm × 30 cm × 20 cm and were built by parallel strands in order to have sufficient width to cut prisms for testing (see Figure 2). The height of the specimen corresponds to 20 layers of 1 cm height each. The time gap between the layers was usually 60 s. In order to increase the layer bonding for all elements (with and without fibers), the layers had a rectangular cross-section of approx. 10 mm × 45 mm and were interlocked comparable to a tongue and groove system (see Figure 2). To this end, the layer height was defined by adjusting the height of the robot arm. Thus, the material of the subsequent layer was pressed slightly into the antecedent layer. The nozzle opening was 11 mm × 42 mm (see Figure 2).

Figure 1 gives an overview on the investigated reinforcement types and whether they were produced with reference material A or B. The placement of the reinforcement varied in dependence of the reinforcement type (see top to bottom in Figure 1):

The micro steelfibers were integrated in the mixing regime of the lightweight mortar and extruded with the mortar (A + Fibers).

**Figure 3:**
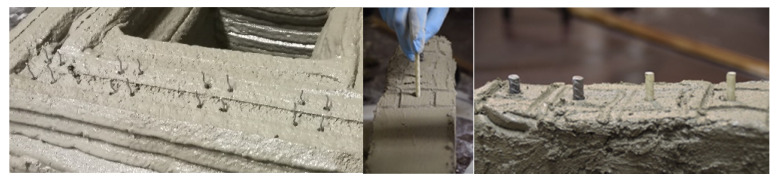
Vertical insertion of reinforcement: wire fibers (**left**), rebars (**middle** and **right**).

The vertical reinforcement was inserted in the freshly deposited layers. The smaller wire fibers were inserted every four layers and had an overlap length of 1 cm. To simulate the insertion of reinforcement in a cold joint area, the time gap was increased to 5 min for the layers, where the fibers were inserted. The 16 wire fibers were placed manually in predetermined intervals of 1.6 mm and 4.8 mm between the layers in order to be able to cut four prisms (see Figure 3, left). The extrusion process was stopped shortly before the next layer was deposited. In this phase, the material was conveyed further and disposed separately.

The larger vertical reinforcements (steel and glass fiber rebars) were inserted after deposition of all 20 layers approx. 20 min after deposition of the first layer, see Figure 3, middle and right. Three rebars each were placed at intervals of 50 mm in order to be able to cut prisms of 40 × 40 × 160 mm^3^. The tests are based on reference material B.

The horizontal reinforcement was placed directly during the extrusion process in the outer strand when one layer was finished and before the next layer was deposited (see Figure 2, middle). It was placed each time after two layers had been deposited. The tests were conducted with reference material A and B.

For comparison, reference specimen without reinforcement were produced in addition to the reinforced lightweight mortar specimen.

### 2.5. Mechanical Tests

**Figure 4:**
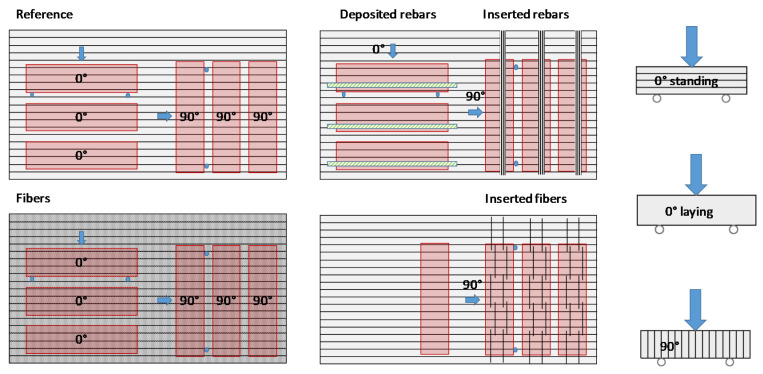
Schema of test prism cut out of 3D-printed element and test directions for flexural strength.

After hardening, test specimen (prisms with 40 mm × 40 mm × 160 mm) of different orientations (0° and/or 90° to the deposition direction, see Figure 4) were sawn out from the sample geometries.

The storage of all prisms took place in a climate chamber at 20 ± 1 °C and 65% ± 5% relative humidity. After 7 days, they were tested with respect to their flexural and compressive strength according to the standard EN 196-1.

The 90° sawn-out prisms are more affected by the layer bonding [13,21]. Thus, they serve to determine the effect of the reinforcement system on the anisotropy. The orientation of the prisms for the tests is shown in Figure 4. For each test series, the flexural strength was investigated on three prisms and the compressive strength on six cubes with edge length 40 mm each. The effect of the vertical reinforcement was only tested on 90° sawn-out prisms (three prisms each) as they are suspected to influence the layer bond.

**Figure 5:**
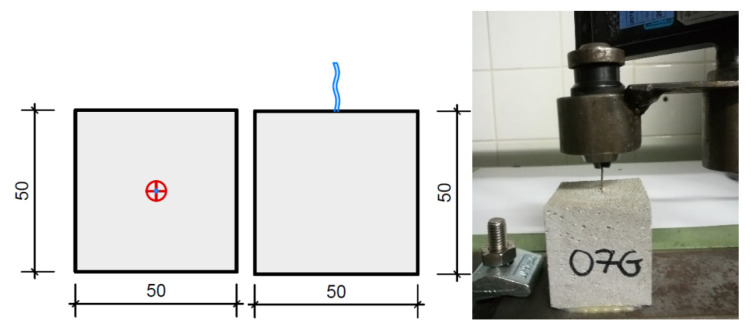
Pull-out tests of the wire fibers: schematic overview (**left**) and sample (**right**).

In addition, the vertical wire fibers were subjected to pull-out tests in order to evaluate the bond between the reinforcement and the lightweight mortar. To this end, samples with five layers were extruded and individual wire fibers were inserted to a depth of approximately 3.5 cm. Cubes with dimensions of 50 × 50 × 50 mm were sawn out (see Figure 5) and the length of the protruding fiber was measured. Thereby, we were able to calculate the bond length.

A spindle-driven universal testing machine with a shaft load cell of up to 500 N was used as testing facility. The test specimens were clamped into the machine and a continuous force was applied to the projecting fiber. The test was carried out as a centric tensile test and was driven force-controlled at 5 N/s. The limitations of the test were either the achievement of 480 N or a machine path of 10 mm. During the test, the force applied was recorded as a function of time and relative displacement between the fibers and the mortar matrix. The bond stress was calculated over the surface of the wire fibers in the bond area and the applied force.

## 3. Results and Discussion

### 3.1. Horizontal Reinforcement

The focus in this paper lies on the mechanical properties, and pumpability is seen as a prerequisite but will not be described in detail. However, the material must be suitable for extrusion. In this section, we want to compare the characteristics of the samples with micro steelfibers extruded with the lightweight mortar and the deposition of horizontal reinforcement elements between two layers with unreinforced samples. In order to be able to extrude the material, it has to fulfill the requirements for pumpability. The pumpability was evaluated by the pressure, flow rate, time in the hose and density before and after pumping. Furthermore, the amount of fibers was investigated before and after pumping. The material was comparably pumpable with and without fibers in a progressive cavity pump and through a 5 m long hose with a diameter of 25 mm. The amount of steel fibers was the same within the accuracy of the test before and after pumping. The cleaning of the hose, however, still needs further investigation when steel fibers are used as rinsing with water does not exert sufficient pressure and fiber nests might occur in the hose.

**Figure 6:**
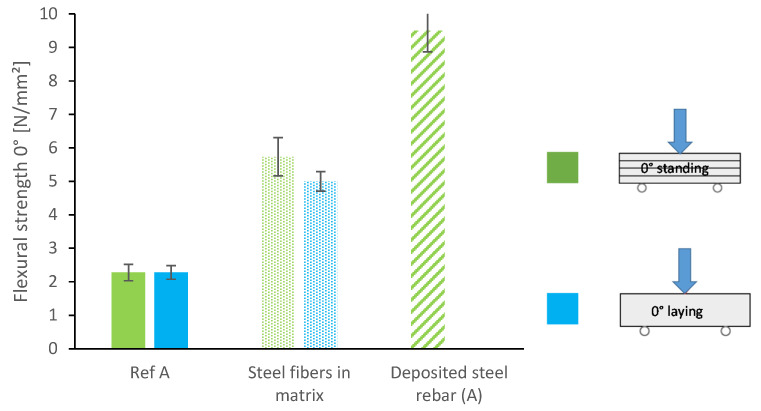
Flexural strength of lightweight mortar specimen: reference, with micro steelfibers and with deposited steel rebar (cut out 0° to printing direction, tested standing (**green**) and laying (**blue**).

Figure 6 depicts the flexural strength of prisms sawn out 0° to the deposition direction of the reference mixture, the mixture with micro steelfibers and the sample with a deposited steel rebar between the layers. In order to account for directional dependencies, we also tested the prisms laying (blue) and standing (green) as schematically demonstrated in Figure 4. We found no significant difference between laying and standing samples, since in both cases the layer bonding extends over the entire width under stress. The flexural strength can be significantly increased by the integration of reinforcement: The steel fibers lead to an increase by 130% on average. The deposited steel rebar even increases the flexural strength by 320%.

Investigations with reference material B showed the same trend. An increase of up to 600% was reached by the horizontally deposited steel rebars (tested standing, see Figure 4) (11.9 N/mm^2^ instead of 1.9 N/mm^2^).

**Figure 7:**
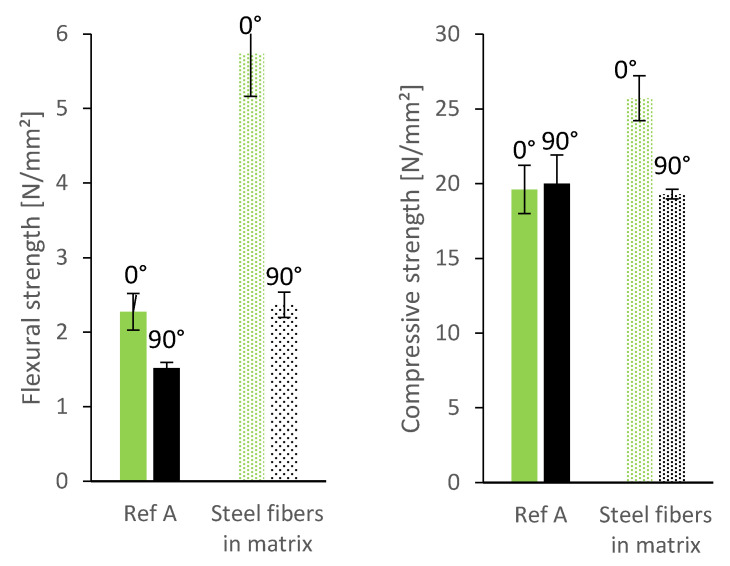
Flexural and compressive strength of specimen without and with micro steelfibers (cut out 0°/90° to printing direction).

**Figure 8:**
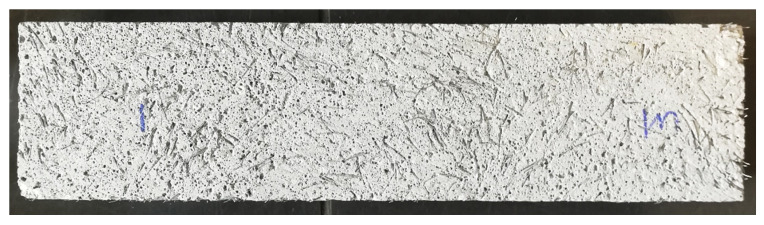
Micro steelfiber orientation in a prism sawn out from a 3D-printed object (side view).

Furthermore, we tested the flexural strength of prisms sawn out 90° to the deposition direction. As described earlier, the layer bonding has a more pronounced effect on the flexural strength when the layer bonding is parallel to the applied force (which is the case when testing prisms sawn out 90° to the deposition direction, see Figure 4). As can be seen in Figure 7, the flexural strength of prisms sawn out at 90° is significantly lower than of prisms sawn out at 0°. The difference between the two directions is even larger for the mixture with micro steelfibers: drop of 3.4 N/mm^2^ (equal 63%) with steel fibers instead of 0.8 N/mm^2^ (equal 33%) for the reference mixture. Still, the flexural strength of the mixture with micro steelfibers at 90° is significantly higher than at 90° for the reference mixture. As the micro steelfibers are deposited with the material, they are suspected not to have an effect on the layer bonding. However, the increase in flexural strength indicates such an effect. This suggests that the steel fibers (see Figure 8) are not oriented as much as in other investigations known from literature (see Section 1.2).

The compressive strength after 7 days is not affected as much by the layer bonding. It is slightly increased by the steel fibers for the prisms sawn out at 0° in comparison to the reference mixture (see Figure 7, right).

### 3.2. Vertical Reinforcement

**Figure 9:**
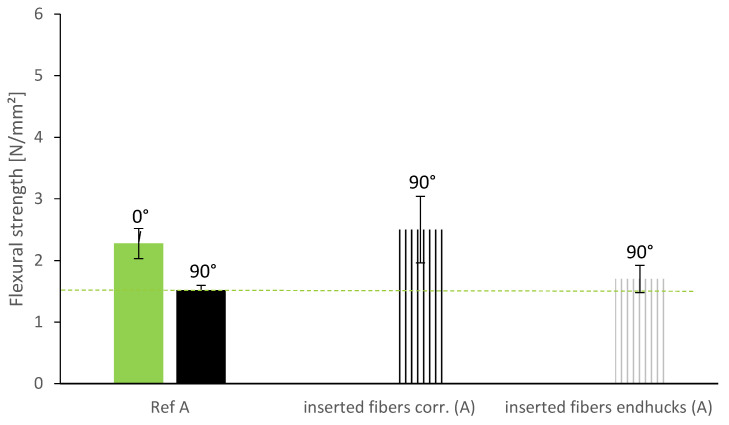
Flexural strength of specimen with vertically inserted fiber reinforcement.

Due to geometrical prerequisites, the layer length might vary within a building element. This leads to varying time gaps between the layers and thus to worse layer bonding in longer layers. In order to compensate the influence of the worsen layer bond, we investigate the insertion of vertical wire fibers. The fibers are inserted vertically into the layer with the larger time gap (5 min instead of 1 min). In Figure 9, we compare the reference mixture produced at constant building rate with the same material with larger time gaps every four layers and compensating reinforcement elements.

The effect of vertically inserted wire fibers depends on the geometry of the fiber. As displayed in Figure 9, the corrugated fibers show a significant average increase of 1 N/mm^2^ (equal to 64% increase) in flexural strength at 90° in comparison to the reference material. The fibers with end hucks, on the other hand, show only a minor increase of 0.2 N/mm^2^ on average. The fibers with end hucks are consequently sufficient to compensate the effect of the irregular building rate. It can be assumed that the wire fibers have no effect on the flexural strength at 0° and therefore have the same flexural strength at 0° as the reference. The corrugated wire fibers reach a flexural strength at 90° that is even larger than the flexural strength at 0°. With this type of fibers, it is thus possible to compensate the anisotropy induced by the layer bonding. The standard deviation of the results indicates that the effect is dependent on the quality of the insertion. We will come back to this matter in Section 3.3.

**Figure 10:**
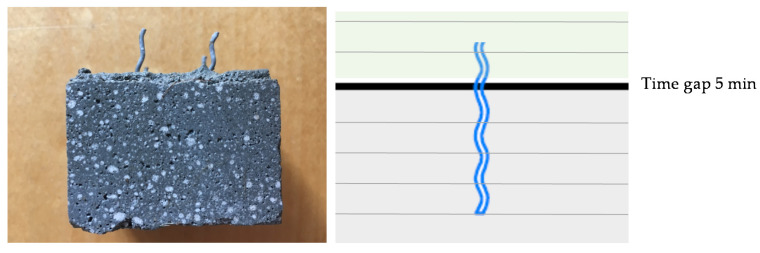
Specimen with vertically inserted fiber reinforcement over cold joints (**left**) and schematically area of fracture (**right**).

The overlap length of the fibers is very short in this test series (1 cm). Thus, the investigated samples failed at the layer bond of the larger time gap and the fibers were pulled out (see Figure 10). The effect of the wire fibers might consequently be increased by increasing the overlap length and thus avoiding the local accumulation of the weak points of mortar and reinforcement.

**Figure 11:**
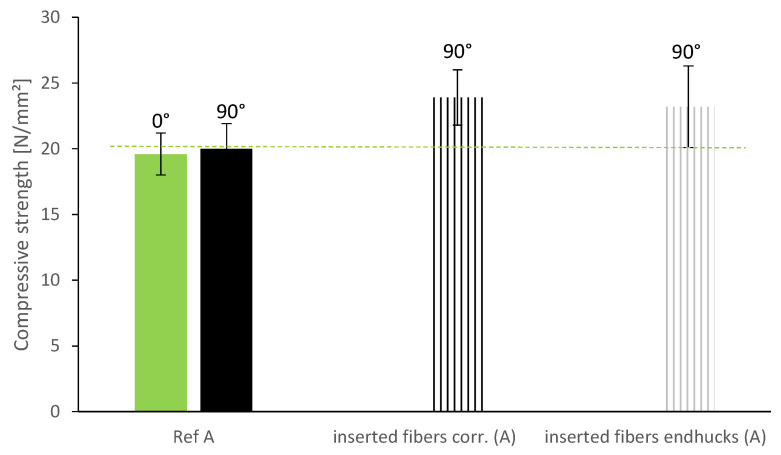
Compressive strength of specimen with vertically inserted fiber reinforcement.

The compressive strength of the specimen is increased by the inserted wire fibers despite the longer time gaps between the layers. Both fiber types show similar values for compressive strength and a minor increase of 3.5 N/mm^2^ (17.5%) on average (see Figure 11).

**Figure 12:**
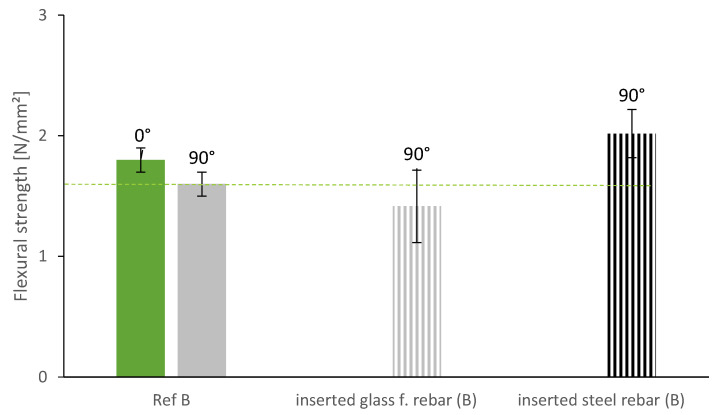
Flexural strength of specimen with vertically inserted rebars.

Furthermore, we investigated the insertion of vertical reinforcement at regular time gaps of 90 s. After the 3D-printed sample was finished at 20 cm, rebars were inserted vertically into the fresh layers. As can be seen in Figure 12, the steel rebars increased the flexural strength and even compensated the effect of the layer bond (higher flexural strength than reference specimen at 0°). The glass fiber rebars on the other hand were not able to increase the flexural strength of the specimen.

The bad performance of the specimen with glass fiber rebars and the comparably low effect of the steel rebars can be traced back to the cavity formation around the rebars in the lower layers. Due to high structural build-up, the yield stress in the lower layers is already significantly higher than in the upper layers and the lightweight mortar is not able to flow around the rebar and ensure a good bound between mortar and reinforcement (see Section 3.4).

### 3.3. Single Fiber Pull-out Tests

**Figure 13:**
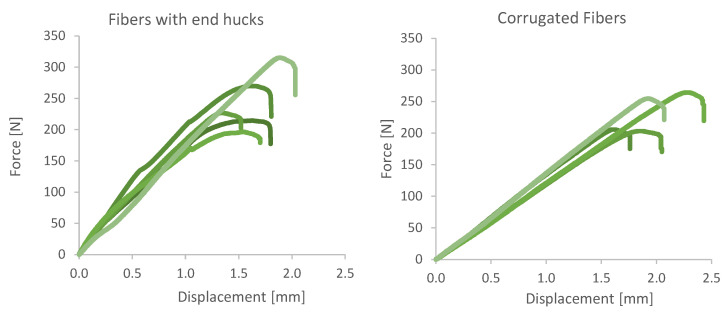
Fiber pullout tests of specimen with vertical fiber reinforcement.

In order to assess the quality of the bond between wire fibers and lightweight mortar, pull-out tests were conducted as described in Section 2.5. The results of the pull-out tests of the wire fibers show a large variation, see Figure 13. This can be attributed to the manual insertion of the fibers and can be eliminated by an automatized fiber insertion using a robotic arm, which can enable straight insertion with better precision. Surprisingly, the fibers with end hucks show comparable bond strength (2.2 N/mm^2^) as the corrugated fibers (2.1 N/mm^2^) even though the latter performed better in the flexural bending tests.

### 3.4. Cavity Formation

**Figure 14:**
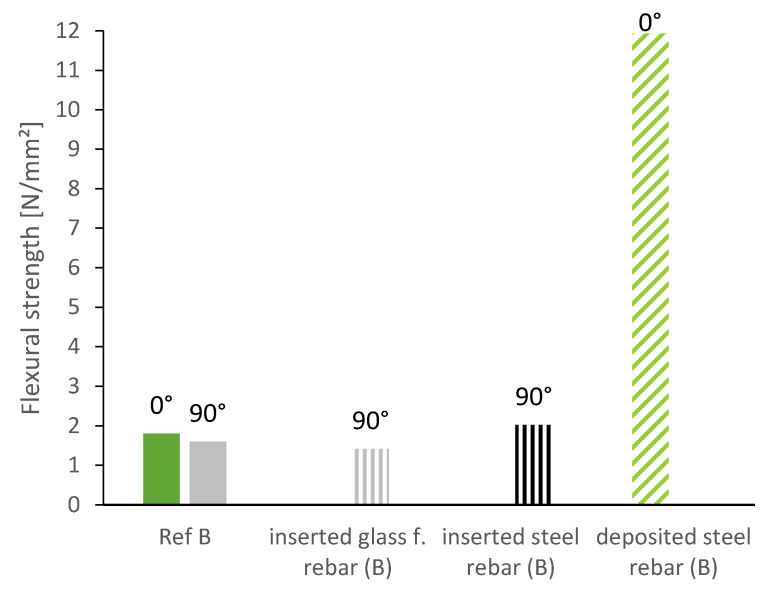
Flexural strength depending on the bond between the rebar (steel/glass fiber) and the mortar in comparison to flexural strength of the reference mixture.

The insertion of vertical elements might lead to cavity formation if the rheological properties of the lightweight mortar prevent a circumvallation of the reinforcement element after insertion. When comparing the horizontally deposited rebars, which were almost directly encompassed by fresh material with the vertically inserted ones, which were inserted into layers of different age, we can see a large discrepancy (11.9 N/mm^2^ vs. 2.0 N/mm^2^, see Figure 14). The flexural strength of the specimen with the horizontal reinforcement element is almost sixfold, compared to the specimen with vertical reinforcement element.

**Figure 15:**
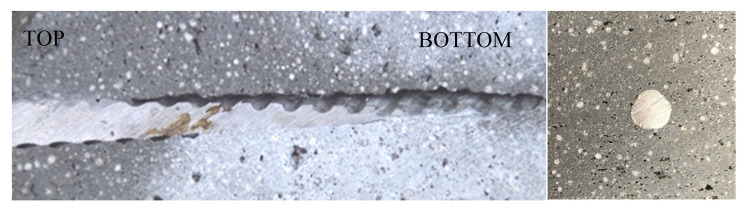
Void formation around the vertically inserted rebar (**left**). Bond between horizontally deposited rebar and mortar (**right**).

**Figure 16:**
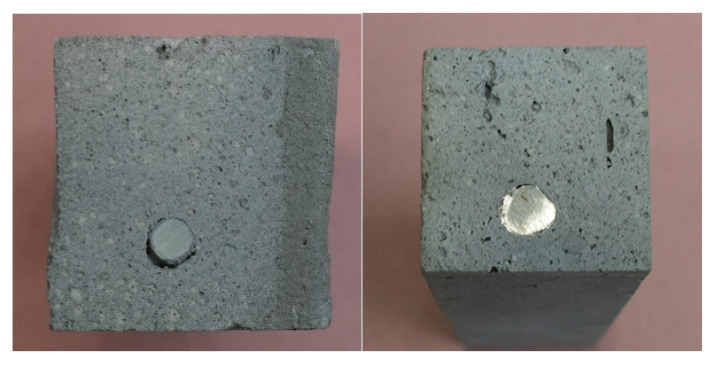
Bond between mortar and rebar: glass fiber (**left**), steel (**right**).

Besides the influence of the layers, the formation of voids or cavities around the vertical reinforcement elements has a significant influence in this matter. The vertical rebars were easy to insert but needed increasing force with increasing depth due to the advanced structural build-up in the lower layers. The bottom two layers were already too hard to insert the rebars manually after 20 min without blurring. The connection between rebar and lightweight mortar varied significantly over the height of the element. Void formation can be observed around some of the inserted vertical elements, especially in the lower layers. Figure 15 shows the profile of a vertically inserted rebar in the lightweight mortar. The horizontally deposited rebar on the other hand has a good bond with the mortar (see Figure 15 right). In Figure 16 we see that the void formation is stronger for the glass fiber rebars.

On basis of the results with the elements that were deposited between two fresh layers and the detection of cavities especially in the lower layers, it can be assumed that the effect of vertically inserted steel elements can be significantly increased by rheological modification of the mortar. This might be by, e.g., setting a softer consistency and ensuring that the mortar is able to flow around the rebar over a longer period of time by changing its structural build-up. Furthermore, it might be useful to vibrate or twist the rebars during insertion in order to activate the thixotropic behavior of the extruded mortar so that the mortar flows around the rebar. This methods lead to an enhanced bond performance between the reinforcement and the surrounding mortar which increases the bond strength itself. Thus, further research is needed in order to exploit the full potential.

**Figure 17:**
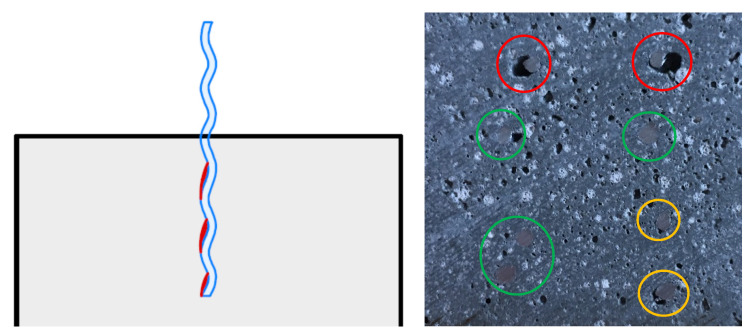
Schematical cavity formation around the vertically inserted fibers (**left**). Different quality of bond between fibers and mortar (**right**—red: bad, yellow: satisfactory, green: good bond behavior).

Figure 17 shows the formation of voids for the corrugated fibers. It varies depending on the position of the fiber in the mortar and on the manual handling. Furthermore, turning the fiber after insertion might lead to a better bond [29]. This might be implemented into the robotic application. Additionally in this case, the adaption of the rheology of the mortar should be investigated.

**Figure 18:**
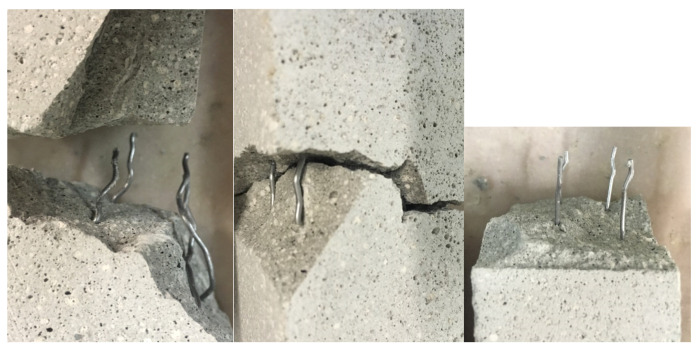
Fracture surface depending on the bond behavior.

The formation of cavities primarily influences the bond between reinforcement and mortar and thus the results of the pull-out tests (see Section 3.3). The variation in flexural strength (see Section 3.2) is furthermore affected by the overlap length of the fibers and the position relative to the layer bond. The fracture surface varies depending on the quality of the bond, the overlap length of the fibers and the position of the layer bond (Figure 18).

## 4. Overarching Discussion

The reinforcement techniques presented are suitable to be integrated in the 3D-printing process. It was shown that the transmission of flexural loads can be enhanced and the effect of cold joints, as described e.g., in [5,6], reduced. As described in Section 1.2, the layer bond often represents a weakness zone in extrusion [22,23]. This is especially true for the production of larger building elements or the manufacturing of houses at side, where the time gap between layers increases and additionally, a need to pause the printing process might occur.

The working hypothesis that vertical reinforcement increases the flexural strength in the zone of the layer bond is supported by the presented results. This is in line with the findings of recently published investigations [25,27,29]. Comparing the results with the literature, we see that the insertion of reinforcement into 3D-printed elements is promising for various types of concrete and additive manufacturing techniques: shotcrete 3D-printing [29], extrusion of normal concrete [27] and extrusion of lightweight mortar as discussed in this paper. The corrugated fibers lead to an increase in flexural strength by 64% while the nail investigated by [25] increased it in the order of 50%.

However, it must be noted that the effect of vertical reinforcement is dependent on the quality of the bond between reinforcement and mortar. Especially, the formation of cavities around vertically inserted elements and possibilities to avoid them must be further investigated. The formation of voids represents a limiting factor. It can be avoided by varying the insertion technique or by adapting the rheology of the mortar. Possible solutions might be the screwing of the reinforcement or the usage of a grouting mortar [29]. However, for the screwing technique robots must be designed for controlled material feed, turning and insertion. This might limit the possible length of reinforcement elements and needs further investigation. If a grouting mortar is used, the bond between extruded mortar and grouting mortar must be investigated. Furthermore, a second device for mixing and providing of the grouting mortar must be developed, taking into account the open time of the grouting mortar. Furthermore, it might be useful to vibrate or twist the rebars during insertion in order to change the rheology of the mortar. However, the effect on the accuracy and deformation of the 3D-printed object must be considered. This is also the case at other variation of the rheology in order to ensure a good bond between reinforcement and mortar—we are touching the conflict between high structural build-up for buildability and low structural build-up for (layer) bonding [24]. The layer bond can be further enhanced by combining the geometrical interlocking of the layers [24] with the application of vertical reinforcement. Additionally, for the rebars, pull-out tests on specimen with varying rheological properties of the mortar are promising in order to assess and increase the bond of the rebars and the mortar.

In addition to the increase in flexural strength, we found that the insertion of small vertical elements is useful to reduce the effect of cold joints. The insertion of wire reinforcement might be a solution to enhance the joint apart from or additionally to the application of glue. In order to investigate this matter, further flexural tests in combination with tensile bond tests should be conducted. In this context, the overlap length, variation of time gaps and insertion depth should be investigated as well.

Applying the fibers with the mortar matrix enhanced the flexural strength of the 3D-printed element parallel to the oriented fibers. This and the dependence on the test direction due to the layer bonding is in line with the findings of [12,13]. Testing prisms sawn out at 90° to the print direction the flexural strength could be increased in comparison to the flexural strength of the reference mixture without fibers. This might be due to the fact that the fibers were not aligned all in the same direction and were able to enhance the layer bonding. The fiber orientation and its deliberate use should be further investigated.

In the present paper the feasibility of the individual reinforcement systems is shown. The costs of the different reinforcement systems vary, e.g., the costs for microfibers are much higher than for rebars in order to achieve the same effect. In future investigations the reinforcement is to be laid out statically effective for all reinforcement types equally which allows an economic estimation of the efficiency of the different reinforcing strategies.

All in all, the results show promising possibilities for the increase of the flexural strength of extruded mortar, especially combined with results from literature and further research.

## 5. Conclusions

In the paper at hand, different reinforcement methods for the extrusion of lightweight mortar were investigated. By far the highest strength results were achieved with reinforcement bars made of steel as horizontal reinforcement. However, due to their limited formability, they significantly limit the freedom of form of the extruded objects. The micro steelfibers in lightweight mortar also achieved high flexural strength and were easy to pump and extrude. The micro steelfibers also increased the flexural strength of prisms sawn out at 90° to the deposition direction to a level of the reference prisms at 0°. This suggests that they are not aligned unidirectionally and that they have an influence on the layer bond, which is favorable for the desired application. However, the subsequent cleaning of the hose caused problems, resulting in a loss of time and thus inefficiency of the entire printing process. The corrugated wire fibers as vertical reinforcement visibly increased the layer bonding and present a potential to compensate cold joints. Larger, vertically inserted steel rebars showed an enhancement of the flexural strength but did not fulfill the expectations. The lower effectiveness in comparison to horizontal reinforcement is due to the formation of cavities during insertion. If the rheology is developed accordingly to improve the bond between reinforcement and mortar or the insertion technique is adapted, a great potential of this technique can be expected.

To conclude, the combined use of fibers in the matrix and vertical and horizontal reinforcement according to necessity is a promising way to extend the application range of (lightweight) concrete extrusion.
